# Expression of Estrogen Receptor α 36 (ESR36) in the Hamster Ovary throughout the Estrous Cycle: Effects of Gonadotropins

**DOI:** 10.1371/journal.pone.0058291

**Published:** 2013-03-29

**Authors:** Prabuddha Chakraborty, Shyamal K. Roy

**Affiliations:** 1 Departments of Cellular and Integrative Physiology, University of Nebraska Medical Center, Omaha, Nebraska, United States of America; 2 Department of Obstetrics and Gynecology, University of Nebraska Medical Center, Omaha, Nebraska, United States of America; 3 Olson Center for Women's Health, University of Nebraska Medical Center, Omaha, Nebraska, United States of America; Baylor College of Medicine, United States of America

## Abstract

Estradiol-17β (E) plays an important role in ovarian follicular development. Evidence indicates that some of the effect of E is mediated by the transmembrane estrogen receptor. In this study, we examined the spatio-temporal expression of recently discovered ERα36 (ESR36), a splice variant of Esr1 and a receptor for non-genomic E signaling, in the hamster ovary during the estrous cycle and the role of gonadotropins and ovarian steroid hormones in ESR36 expression. ESR36 expression was high on estrus (D1∶0900 h) and declined precipitously by proestrus (D4∶0900 h) and remained low up to D4∶1600 h. Immunofluorescence findings corroborated immunoblot findings and revealed that ESR36 was expressed only in the cell membrane of both follicular and non-follicular cells, except the oocytes. Ovarian ESR36 was capable of binding to the E-affinity matrix, and have different molecular weight than that of the ESR1 or GPER. Hypophysectomy (Hx) resulted in a marked decline in ESR36 protein levels. FSH and LH, alone or combined, markedly upregulated ESR36 protein in Hx hamsters to the levels observed in D1 hamsters, but neither E nor P had any effect. Inhibition of the gonadotropin surge by phenobarbital treatment on D4∶1100 h attenuated ESR36 expression in D1∶0900 h ovaries, but the decline was restored by either FSH or LH replacement on D4 afternoon. This is the first report to show that ESR36, which is distinct from ESR1 or GPER is expressed in the plasma membrane of ovarian follicular and non-follicular cells, binds to E and its expression is regulated directly by the gonadotropins. In light of our previous findings, the results suggest that ovarian cells contain at least two distinct membrane estrogen receptors, such as GPER and ESR36, and strongly suggest for a non-genomic action of E regulating ovarian follicular functions.

## Introduction

Estradiol-17β (E) plays a key role in mammalian folliculogenesis [1], [2]. E stimulates the formation of gap junctions [3] and proliferation of granulosa cells (GC), and also enhances the action of FSH and LH in the ovary [4]. The genomic action of E is mediated by classic estrogen receptor α (ESR1) and estrogen receptor β (ESR2). αERKO mice are acyclic and infertile with hemorrhagic and cystic antral follicles. High level of serum LH in these animals is one of the major causal factors in the formation of abnormal, non-ovulatory antral follicles [5]–[7]. In contrast, the infertility in βERKO mice is due to defects in follicular cells [8].

Evidence indicates that E can signal rapidly through a non-genomic pathway in many cell types including those from the reproductive organs [9]–[14]. Membrane estrogen receptors have been shown to activate different signaling pathways, such as adenylate cyclase [15], phospholipase C (PKC) [16] or mitogen activated protein kinase (MAPK) [17]. Membrane localized ESR1, ESR2 or G-protein coupled estrogen receptor 30 (GPER) has been shown to transduce estrogen signaling in cancer and murine ovarian cells [18]–[20]. Recently, a shorter variant of ESR1, ESR36 has been shown to mediate the non-genomic estrogen signaling in breast cancer cell lines [21], [22].

Esr1 gene gives rise to a full-length and several alternately spliced transcripts in all species examined. In the hamster ovary, Esr1 gene is transcribed into one full-length and two alternately spliced transcripts of smaller size [23]. ESR36 is an alternatively spliced truncated form of the full length Esr1, originates from a promoter located in the first intron of the Esr1 gene, and is devoid of both transactivation domains while retaining the DBD and partial LBD and receptor-dimerization domain [24]. Lack of transcriptional co-activation domains implies that ESR36 cannot transduce E signaling through conventional genomic pathway like ESR1. Accordingly, it may act as a transmembrane receptor to mediate the non-genomic action of E in estrogen responsive cells including those in the ovary. In fact, E modulates signaling pathways including PI3K/Akt and MAPK in many classic ESR-free cell lines overexpressing ESR36 transgene [22], [25], [26]. Because the promoter for ESR36 is different from that of the Esr1 it is likely that different mechanisms may regulate ESR36 expression especially in ovarian cells. Using primarily immunofluorescence localization, ESR36 has been detected in postnatal mouse ovaries [27]; however, the cellular localization remains obscure. Further, virtually nothing is known about the spatio-temporal expression or hormonal regulation of ESR36 in ovarian follicular and non-follicular cells with respect to the estrous cycles. The objectives of the present study were to delineate whether ESR36 was expressed in hamster ovarian cells in an estrous cycle dependent manner and whether the expression was affected by FSH, LH, E or progesterone (P). We selected golden hamsters based on the precise nature of their estrous cycles, well-defined stages of follicular development and serum levels of reproductive hormones corresponding to the estrous cycles, and our earlier data on the expression of ESR [23] and GPER [19] in the ovary during the estrous cycles.

## Materials and Methods

An antibody against the C-terminal region of the ESR36 protein was kindly provided by Dr. Z.Y. Wang (Creighton University Medical center, Omaha, Nebraska). The antibody was thoroughly characterized for its specificity using various cell lines [21], [22] and mouse ovaries [27] in which the antibody did not cross-react with the ESR. Peroxidase-conjugated (for immunoblotting) and a DyLight-488-conjugated (for immunofluorescence) secondary antibodies were obtained from Jackson Immunoresearch, Inc. (West Grove, PA); chemiluminescence detection kit (ECL Advance) was obtained from GE Healthcare (Piscataway, NJ); Optitran transfer membrane (Schleicher & Schuell Biosciences, Dassel, Germany) was obtained from Midwest Scientific, Inc. (St. Louis, MO). All other molecular biology grade chemicals were obtained from Sigma Chemical Co. (St. Louis, MO), or United States Biochemical (Cleveland, OH) or ThermoFisher Scientific Corp. (Pittsburgh, PA). Sodium phenobarbital (65 mg/ml), estradiol-cipionate (E, Upjohn, Kalamazoo, MI) and P were purchased from the University of Nebraska Medical Center pharmacy. Ovine-FSH-20 and Ovine-LH-25 were purchased from Dr. A. F. Parlow, Harbor UCLA Medical Center (the National Pituitary Hormone Program, NIH).

Female golden hamsters (90–100 grams body weight) were obtained from Harlan Sprague Dawley Laboratories (Madison, WI), housed in climate-controlled environment with 14 h light and 10 h dark cycle, and given free access to food and water. The study was carried out in strict accordance with the guidelines of the United States Department of Agriculture and the Institutional Animal Care and Use Committee (IACUC) of University of Nebraska Medical center. The use of animals in this protocol was in accordance with the IACAC approval (Permit number: 95-052-03). All surgeries were done under Nembutal anesthesia according to veterinary guidelines, and IACUC approved pain control protocols were used to eliminate post-operative pain and discomfort.

Experiment 1: Ovaries were obtained from hamsters with at least three consecutive estrous cycles at 0900 h on each day of the estrous cycle and at 1600 h on proestrus (after the gonadotropin surges on Day 4). Three animals were used for each day. The experiment was repeated three times.

Experiment 2: Hamsters were hypophysectomized at D1∶0900 h (estrus) using previously described protocol [28], [29]. On post-operative day 10, hamsters were divided into eight groups each containing three animals. Hamsters in groups 1 through 4 were injected sc twice daily at 0900 h and 1600 h with 100 µl of either (1) 0.5% BSA in saline (vehicle for protein hormones), or (2) 10 μg ovine-FSH-20 (NIDDK-NIH) for two days, or (3) 5 μg ovine-LH-25 (NIDDK-NIH) for two days, or (4) 10 μg FSH and 5 μg LH injected at different sites. These doses of gonadotropins were used in many experiments, and they produced physiological responses [30]–[32]. Ovaries were retrieved 48 h after the first hormone injection at 0900 h.

Hypophysectomized hamsters in groups 5 through 8 were injected sc at 0900 h with a single dose of (1) sesame oil for vehicle control, or (2) 100 μg E, or (3) 500 μg P, or (4) a combination of E and P. Ovaries were collected 24 h after the injection. Ovaries from each animal were processed separately as one sample. The entire experiment was repeated twice.

Experiment 3: Nine cyclic hamsters were treated sc with phenobarbital (10 mg/kg body weight diluted in sterile saline) [33] at D4∶1100 h to block the preovulatory LH and FSH surges. As reference controls, ovaries were collected from three untreated hamsters at D1∶0900 h and three untreated hamsters at D4∶0900 h. Ovaries from each animal were processed separately. To mimic the effect of the FSH or LH surge, three phenobarbital-treated hamsters were injected sc with 0.5% BSA in saline (vehicle), three were treated with 10 μg ovine-FSH-20 at 1500 h, and three were treated with 5 μg ovine-LH-25 at D4∶1400 h. Ovaries from all animals were collected at D1∶0900 h and ovaries from each animal was processed separately as individual samples. Ovaries were either embedded in Optimum Cutting Temperature (OCT) medium for cryosections or flash-frozen in liquid N_2_ for protein extraction, and kept at –80°C until use. The entire experiment was repeated twice.

### Western blot analysis of ESR36 protein in the ovary

Ovaries were homogenized by Omni 2000 homogenizer in 50 mM HEPES, pH 7.4, containing 100 mM NaCl, 1 mM EDTA, 1 mM EGTA, 1 mM NaF, 20 mM Na_4_P_2_O_7_, 1% Triton X-100, 10% glycerol, 0.1% SDS, 0.5% deoxycholate, 10% protease inhibitor cocktail (Sigma) and 200 mM Na_3_VO_4_ on ice. The homogenate was centrifuged at 15000× g for 30 minutes at 4°C and the supernatant was used for protein estimation by BCA method (Pierce, Rockford, IL).

Subcellular fractionation was done by homogenizing hamster ovaries collected at Day 3∶0900 h in 50 mm Tris-HCl buffer (pH 7.0) containing 150 mM NaCl, 1 mM EDTA, 1 mM EGTA, 1 mM NaF, 20 mM Na-pyruvate, 2 mM Na_3_VO_4_, 10% glycerol, and 10% protease inhibitor cocktail on ice using a Dounce homogenizer. The homogenate was centrifuged at 1,000× g for 15 mins at 4°C, and the supernatant was further centrifuged at 100,000× g for 1 h at 4°C. The supernatant was used as the cytosolic fraction (C) while the pellet was sonicated in 10 mM Tris-HCl buffer, pH 7.4, containing 100 mM NaCl, 1 mM EDTA, 1 mM EGTA, 1 mM NaF, 20 mM Na_4_P2O_7_, 2 mM Na_3_VO_4_, 1% Triton X-100 and 10% protease inhibitor cocktail, and centrifuged at 15,000× *g* for 30 min at 4°C. The supernatant was used as the crude membrane fraction (M). The 1,000× g pellet was sonicated in 10 mM Tris-HCl buffer, pH 7.4, containing 100 mM NaCl, 1 mM EDTA, 1 mM EGTA, 1 mM NaF, 20 mM Na_4_P_2_O_7_, 2 mM Na_3_VO_4_, 1% Triton X-100 and 10% protease inhibitor cocktail, and centrifuged at 15,000× *g* for 30 min at 4°C. The supernatant was used as the nuclear fraction (N).

Forty micrograms whole ovary homogenate, 40 μg subcellular fractions and 2 ng recombinant human ESR1 were resolved in 12% polyacrylamide gels along with the Precision Blue molecular weight markers (Bio-Rad), transferred to Optitran nitrocellulose membrane, blocked with 5% non-fat dry milk in TBST and probed with the ESR36 antibody in 5% non-fat dry milk in TBST overnight at 4°C. After washing, the membrane was probed with appropriate second antibody conjugated to peroxidase for 1 h at room temperature, rinsed and exposed to Advanced Western Blotting detection kit (ECL). The chemiluminescence signal was digitized by the UVP gel documentation system (UVP, Upland, CA). Each membrane was also probed with an antibody against Na-K-ATPase (membrane protein), GPER (membrane protein), ESR1 (nuclear protein) or glyceraldehyde 3-phosphate dehydrogenase (GAPDH) to verify the purity of the subcellular fractions as well as to characterize the specificity of the ESR36 antibody. The rationale for probing the samples with ESR36, GPER and ESR1 antibodies was to determine if those three proteins represented the same receptor protein in ovarian cell membrane. For further validation of the specificity of the ESR36 antibody, 40 μg ovarian protein from hamsters at Day 3∶0900 h was Western blotted with the ESR36 antibody to detect the ESR36 protein band. Then the membrane was probed without stripping with the ESR1 specific antibody [23] to determine whether ESR1 and ESR36 were different proteins.

### Immunofluorescence localization of ESR36 protein

Six micron-thick frozen sections were fixed in freshly prepared ice-cold 4% paraformaldehyde in PBS (pH 7.4) and used for localizing ESR36 protein using 1∶1500 dilution of the ESR36 antibody. The signal was developed using donkey antirabbit-IgG-DyLight-488 and nuclei were stained with 4′,6-diamino-2-phenylindole. The images were captured by a Leica DMR microscope (North Central Instruments, Plymouth, MN) and Openlab image analysis software (Improvision, Lexinton, MA). The exposure time was set to eliminate any non-specific background signal emitting from sections incubated without the primary antibody, and the signal above background was considered antigen-specific signal. Representative images were organized using Adobe CS5 software without modifying the contrast of the original immunosignal.

### Binding of ESR36 to estradiol-17β (E)

To determine whether ESR36 could actually bind to E, purified plasma membrane was prepared essentially as described by Braun and Thomas [34]. Briefly, the crude plasma membrane was resuspended in 2 ml homogenization buffer without detergent, layered carefully on a 2 ml 1.2 M sucrose cushion, and centrifuged at 6,900 g for 45 min. The membrane fraction at the sucrose-buffer interface was carefully aspirated, diluted 1∶2 with the homogenization buffer and centrifuged at 20,000 g for 20 min. The pellet was sonicated for 5 sec in homogenization buffer containing 1% triton X100, kept on ice for 30 min, and then centrifuged at 20,000 g for 30 min to obtain detergent solubilized plasma membrane proteins including ESR36. The protein concentration was measured by BCA reagent. The triton X100 in 500 µg protein in 100 µl supernatant was removed using a detergent removal column (Pierce) according to the manufacturer's instructions and 80 µl elute was mixed overnight at 4°C with 50 μl slurry of estradiol-17β-sepharose affinity resin (kindly provided by Dr. Geoffrey Greene, University of Chicago) in 300 μl homogenization buffer without detergent. The mixture was centrifuged at 12,000 g for 30 sec, the supernatant removed, the pellet resuspended in 1 ml ice-cold homogenization buffer without detergent, centrifuged for 30 sec at 12,000 g and supernatant removed. The rinsing step was repeated four more times, and the pellet was finally resuspended in 30 μl 3× reducing buffer containing mercaptoethanol, the protein denatured for 15 min at 37°C, and centrifuged at 12,000 g for 1 min. The supernatant and 40 μg of total plasma membrane protein were fractionated in 10% PAGE, transferred to Optitran membrane and probed with the ESR36 antibody as described previously.

### Hormone measurement

E and P levels in the sera from hypophysectomized and phenobarbital treated hamsters were determined by radioimmunoassay using previously published protocols [35]. The sensitivity of P and E assays was 1 ng/ml and 400 pg/ml, respectively. Anti-progesterone antibody had 0.7% cross-reactivity with androstenedione (A), but none with E. Similarly, the anti-E-antibody did not cross react with either P or A. The levels were presented as pg steroid per ml of serum. The interassay and intrassay variation was within 10% and 5%, respectively.

### Statistical analysis of data

Each group in all experiments had at least three animals and each experiment was repeated at least twice. Within each experiment, the mean of each group was compared to each other using one-way ANOVA followed by the Newman-Keuls *post hoc* test using the GraphPad Prism 5 software (Graph Pad software Inc., La Jolla, CA). The level of significance was 5%.

## Results

### ESR36 protein expression in the hamster ovary throughout the estrous cycle

**Figure 1:**
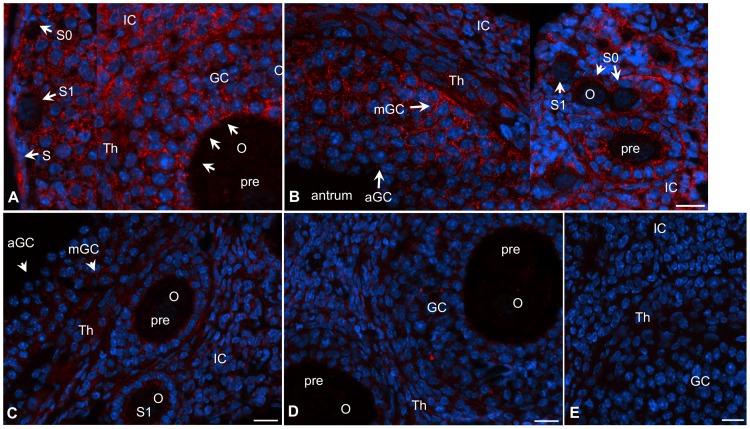
ESR36 is temporally expressed in ovarian cells during the estrous cycles. (A) A section of an ovary at D1∶0900 h showing ESR36 (red) expression in the granulosa (GC), theca (Th) and insterstitial (IC) and surface epithelial (S) cells. (B) A composite of sections of an ovary at D3∶0900 h emphasizing ESR36 gradient in the mural (mGC) and antral (aGC) granulosa cells of antral follicles. Distinct immunostaining was detected in small and large preantral (pre) follicles including primordial follicles (S0). (C) Sections of ovaries at D4∶0900 h and (D) D4∶1600 h showing low levels of ESR36 expression in follicular and non-follicular cells. (E) Section of an ovary at D1∶0900 h exposed to preimmune rabbit serum instead of ESR36 antibody. ESR36  =  red; nuclei  =  blue; S0  =  primordial follicles; S1  =  primary follicles; O =  oocytes; arrowheads  =  granulosa cell processes. Bar  = 10 μm.

The rationale was to localize spatiotemporal expression of ESR36 in the ovary in order to identify a possible role of ESR36 in various cell types. Although ESR36 was detected in the granulosa (GC), thecal (Th) as well interstitial cells (IC), cell-type specific expression was apparent (Fig. 1). ESR36 expression was intense at D1∶0900 h (Fig. 1A) through D3∶0900 h (Fig. 1B), but declined remarkably by D4∶0900 (Fig. 1C) through D4∶1600 h (Fig. 1D). ESR36 was detected in dormant granulosa cells of primordial follicles (S0) and activated granulosa cells of primary follicles (S1) (Figs. 1A and 1B). Granulosa cells of preantral follicles of all stages and granulosa cell processes adjacent to the oocyte showed distinct ESR36 staining (Fig. 1A, arrows). At D3∶0900h, robust ESR36 expression was evident in the mural granulosa cells (mGC) of antral follicles; however, the intensity was somewhat low in the antral granulosa cells (aGC) as well as in thecal cells (Th) (Fig. 1B). Because magnification lower than 200x masked the subtle difference in follicular ESR36 expression, no such image was furnished. Granulosa cells of primordial (S0), primary (S1) as well as small preantral (pre) follicles had strong ESR36 expression (Fig. 1B). By D4∶0900 h, ESR36 immunoreactivity declined sharply throughout the ovary and the decline was most drastic for the mural granulosa cells (mGC) of antral follicles (Fig. 1C). Granulosa cells of primary or preantral follicles had very low levels of expression. ESR36 expression in all ovarian cells decreased further by D4∶1600 h (Fig. 1D). No ESR36 expression could be detected in the oocyte (O) of any follicles or in ovarian cells at D1∶0900 h without the ESR36 antibody (Fig. 1E).

**Figure 2:**
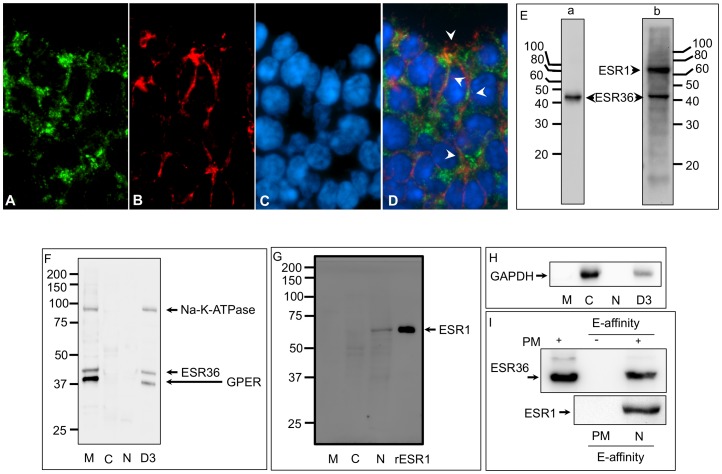
ESR36 in the plasma membranes binds to E and is distinct from ESR1 and GPER. (A–D) Images of the granulosa cell layers of an antral follicle at D3∶0900 h showing (A) ERS36, (B) CDH2, (C) nuclei and (D) a merged image to highlight plasma membrane location (arrowheads) of ESR36. (E) Full length Western blots of ovaries at D3∶0900 h hamster ovary homogenate showing ESR36 (a) and ESR36 and ESR1 (b). The blot was first probed with the ESR36 antibody (a) to determine whether the antibody would detect only one band, which was crucial for its specificity and usefulness in immunofluorescence localization. Then, the blot was probed without stripping with a monoclonal ESR1 antibody (b) to determine whether ESR36 and ESR1 were different proteins. (F) Full length Western blot showing the presence of ESR36 in the membrane fraction (M) and whole homogenate (D3) of ovaries at D3∶0900 h. Distinct molecular weight differences between ESR36 and GPER were also evident. The presence of Na^+^-K^+^-ATPase in the membrane fraction validated the purity of the subcellular fractions. (G) Western blot of subcellular fractions of ovaries at D3∶0900 showing the presence of ESR1 only in the nuclear faction (N). The molecular weight of the hamster ESR1 and recombinant human ESR1 was similar, but was different than that of the ESR36 or GPER (Figs. 2E and 2F). (H) Western blot of subcellular fractions of ovaries at D3∶0900 showing the presence of glyceraldehyde-3-phosphate dehydrogenase (GAPDH) only in the cytosolic fraction, thus verifying the purity of the fractions. (I) ESR36 was present in the sucrose-density gradient purified plasma membrane preparation (PM) and was capable of binding to estradiol-17β cross-lined to sepharose beads (E-affinity). No signal was detected when affinity-beads were incubated without the plasma membrane preparation indicating the specificity of the binding. No ESR1 could be pulled down from the plasma membrane fraction, but the affinity matrix could pull down ESR1 from the nuclear fraction.

We wondered whether ESR36 was localized only in the plasma membrane or elsewhere in ovarian cells. Higher magnification of the granulosa cells of antral follicles in ovaries at D3∶0900 h revealed that ESR36 immunofluorescence was located in the cell membrane (Fig. 2A, C and D) and co-localized with CDH2 (plasma membrane marker) immunostaining (Fig. 2B and 2D). The ESR36 antibody detected a single 43 kDa protein band in ovarian homogenate (Fig. 2Ea) whereas the ESR1 antibody detected an approximately 65 kDa band in the same blot (Fig. 2Eb). Western blot analysis of ovarian sub-cellular fractions with the ESR36 antibody revealed a 43 kDa protein in purified plasma membrane fraction as well as in D3∶0900 h whole ovary homogenate, whereas the GPER antibody detected an approximately 40 kDa protein (Fig. 2F). ESR1 protein could be detected only in the nuclear fraction, but not in the plasma membrane or cytosolic fractions, and the molecular weight corresponded to recombinant human ESR1 (Fig. 2G). The membrane localization of ESR36 was confirmed by the presence of Na-K-ATPase in the membrane fraction (Fig. 2F), ESR1 in the nuclear fraction (Fig. 2G) and GAPDH in the cytosolic fraction (Fig. 2H). The presence of ESR36 in sucrose density-purified plasma membrane fraction further verified its membrane localization (Fig. 2I). ESR36 in purified plasma membranes bound to the E-affinity resin demonstrating the ability of the naturally occurring ESR36 to bind to the E ligand; however, no ESR1 protein could be detected in the same affinity purified samples (Fig. 2I). Converely, ESR1 protein was detected in the E-affinity resin-bound nuclear fraction (Fig. 2I). No ESR36 signal could be detected in the absence of the membrane proteins (Fig. 2I).

**Figure 3:**
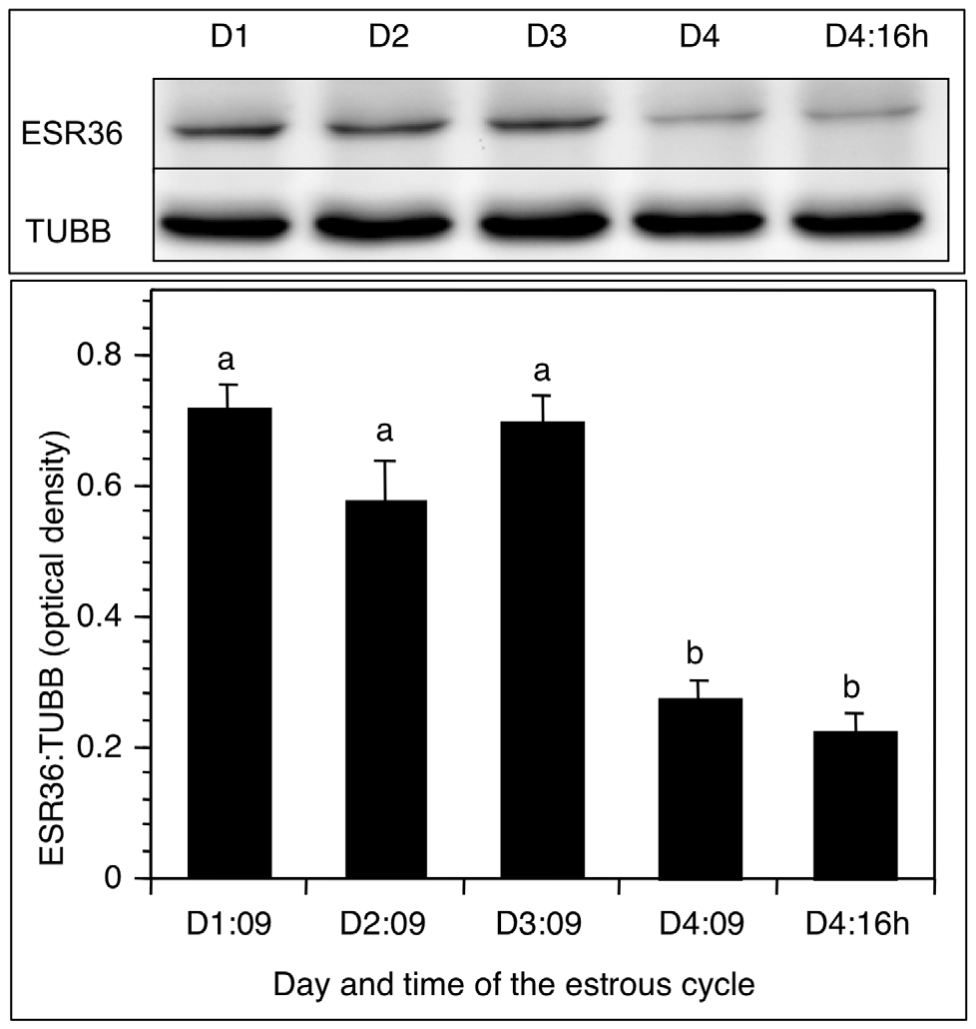
ESR36 protein expression in the ovary declines by proestrus morning (D4:0900h). (Top) a representative immunoblot showing the ESR36 expression in ovarian homogenates, and (bottom) mean optical density ± SEM of the ratio of ESR36:TUBB of three separate samples. *p*<0.05, bars with a different letter.

Immunoblot analysis of ovaries from each day of the estrous cycle and after the gonadotropin surge revealed high levels of ESR36 from D1 through D3∶0900 h (Fig. 3). ESR36 levels declined markedly (*p*<0.05) by the morning of D4 and remained low after the gonadotropin surge (Fig. 3). No change in TUBB expression was observed during the estrous cycles indicating the specificity of changes in ESR36 levels.

### Effect of gonadotropins and steroid hormones on ESR36 expression in the hamster ovary

**Figure 4:**
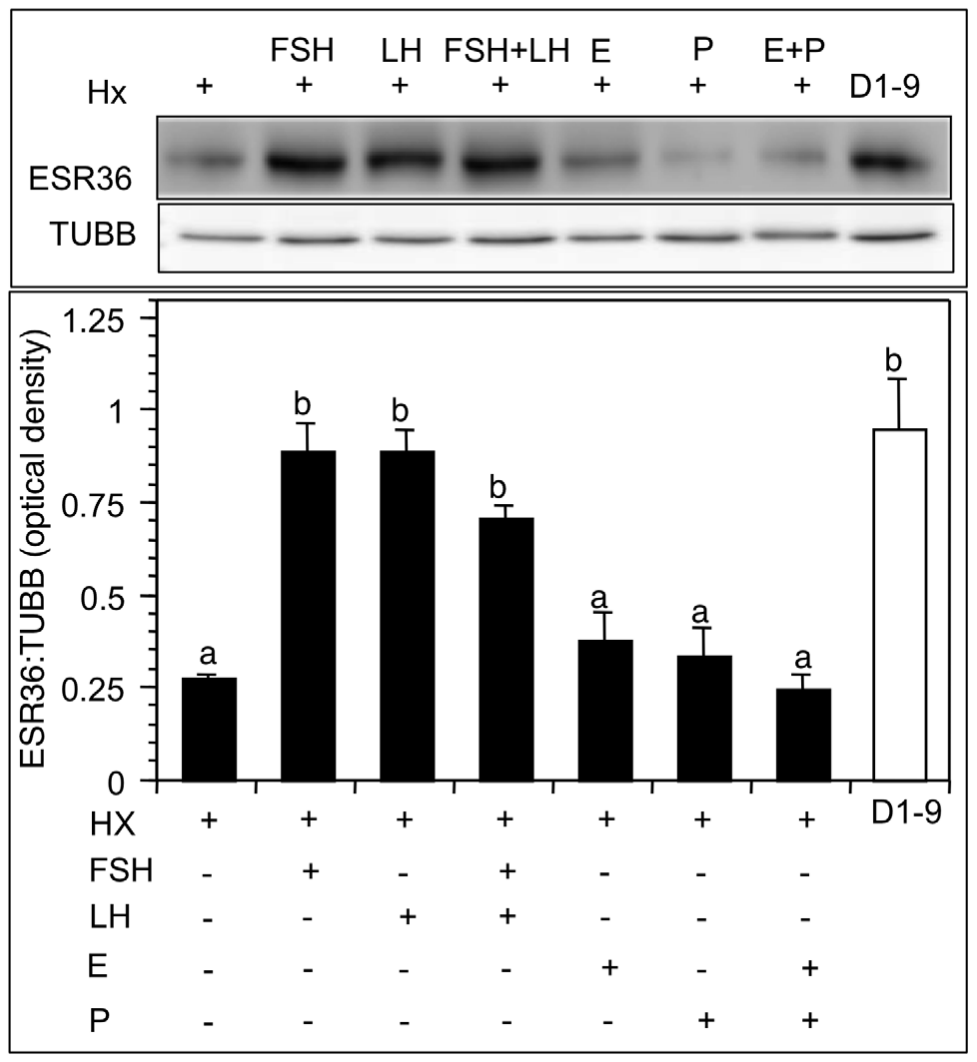
ESR36 expression is upregulated by either FSH or LH, but not by ovarian steroid hormones. Animals were treated sc twice daily for two days without or with FSH, or LH, or FSH + LH, or once with a sc injection of E, or P, or E + P. The levels of ESR36 protein in Hx hamsters were compared with those of intact hamsters at D1∶0900 h because hamsters were hypopysectmized on D1∶0900 h. (Top) a representative immunoblot showing the ESR36 expression in the ovaries, and (bottom) mean optical density ± SEM of the ratio of ESR36: TUBB of three separate samples. *p*<0.05, bars with a different letter.

The rationale was to determine if ovarian ESR36 protein expression was regulated by gonadotropins, either directly or indirectly by ovarian steroid hormones. Hypophysectomy (Hx) at D1∶0900 h resulted 10-days later in a marked reduction (*p*<0.01) in ovarian ESR36 levels compared to the levels observed in hamsters with intact pituitary at D1∶0900 h (Fig. 4). In fact, the values for the Hx hamsters were similar to those observed in ovaries of hamsters at D4∶0900 h (compare with Fig. 3). Treatment of Hx hamsters with FSH or LH for two days fully restored ESR36 expression to the levels observed in hamsters at D1∶0900 (Fig. 4). A combination of FSH and LH also restored the ESR36 expression similar to that observed for either hormone alone (Fig. 4). In contrast to gonadotropins, neither E nor P was able to restore ovarian ESR36 expression (Fig. 4).

**Figure 5:**
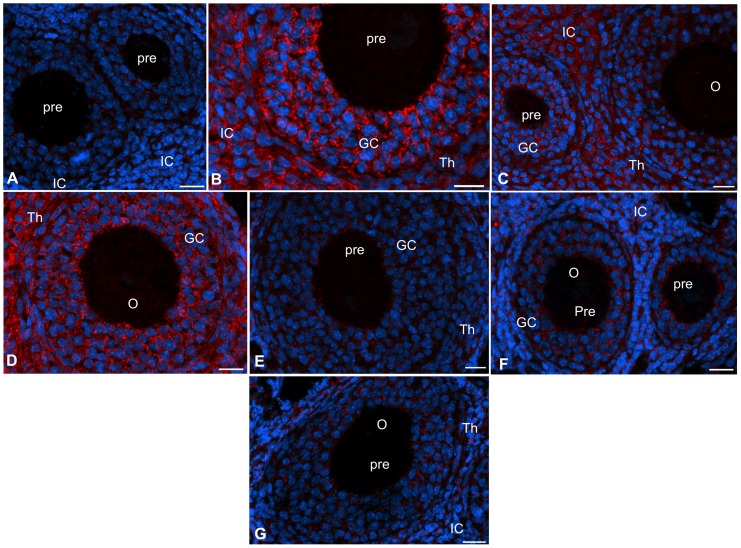
ESR36 expression in the granulosa and thecal cells is regulated by FSH and LH, respectively. Ovaries were collected twelve days after hypophysectomy (Hx) and after selective replacement of FSH, LH, E or P as described in Fig. 4. (A) Hx with saline, (B) twice daily treatment with 10 μg O-FSH-20 for two days, (C) twice daily treatment with 5 μg O-LH-30 for two days, (D) twice daily treatment with 10 μg FSH-20+5 μg LH for two days, (E) one day treatment with 10 μg estradiol-cypionate (E), (F) one day treatment with 10 μg progesterone (P), and (G) one day treatment with 10 μg E+10 μg P. ESR36  =  red; nuclei  =  blue; GC  =  granulosa cells, Th  =  thecal cells, pre  =  preantral follicles, IC  =  interstitial cells, O =  oocytes. Bar  = 10 μm.

**Figure 6:**
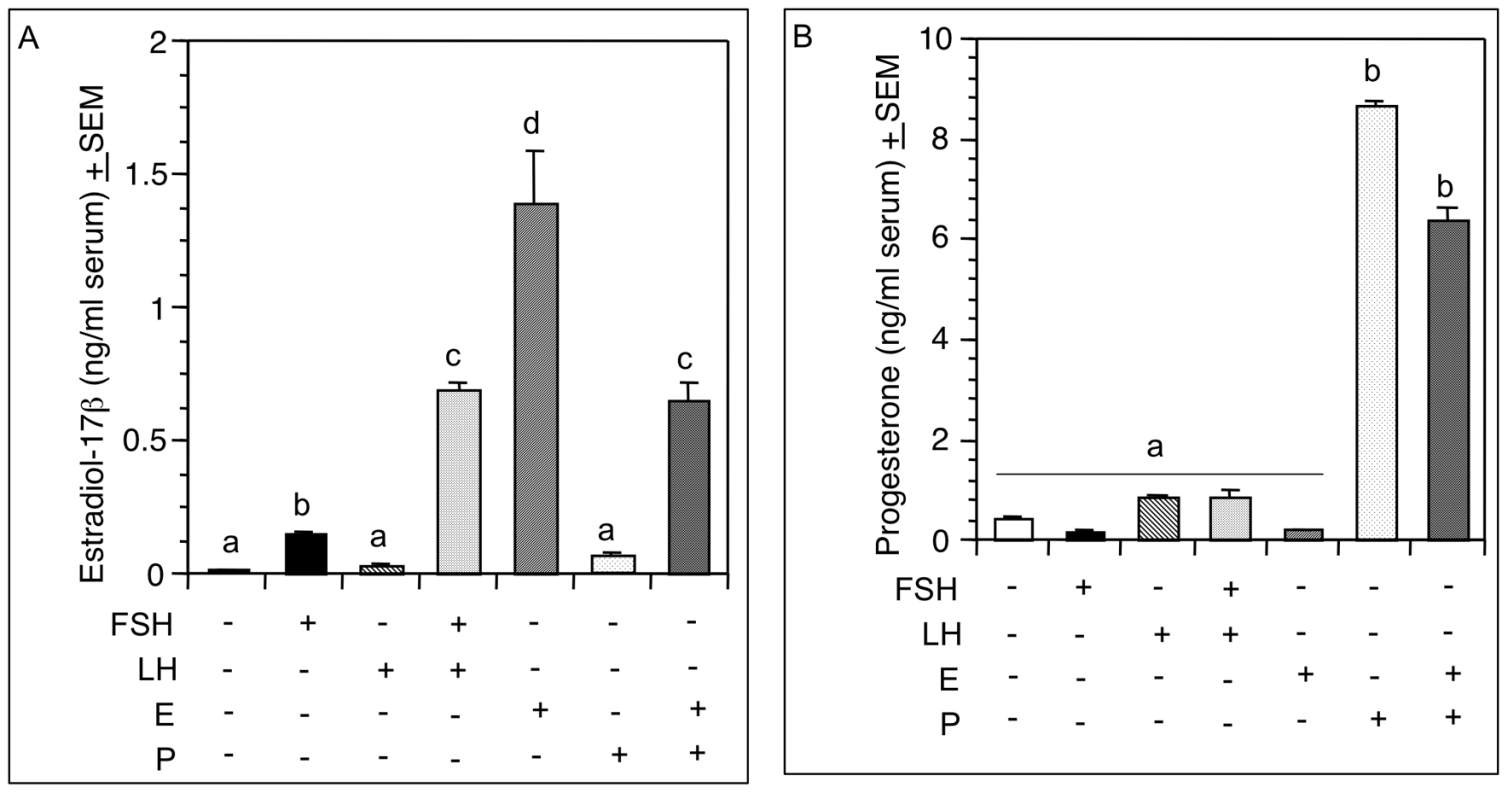
FSH increases serum levels of E. Serum levels of estadiol-17β (A) and progesterone (B) after hypophysectomy and hormone replacement. *p*<0.05, bars with a different letter.

Immunofluorescence findings corroborated the immunoblot data and revealed that ESR36 expression decreased markedly in the granulosa and theca cells of remaining preantral follicles and interstitial cells in the ovaries of Hx hamsters (Fig. 5A). FSH replacement induced the formation of large antral follicles concurrent with increased ESR36 expression in the granulosa cells of antral as well as large preantral follicles (Fig. 5B), but thecal and interstitial cells also had distinct ESR36 immunosignal (Fig. 5B). LH treatment resulted in a marked increase in ESR36 expression mainly in the interstitial cells and theca, but noticeable increase was also evident in the granulosa cells (Fig. 5C). ESR36 expression was prominent in ovaries treated with a combined doses of FSH and LH (Fig. 5D). Neither E (Fig. 5E) nor P (Fig. 5F) alone or combined (Fig. 5G) was able to upregulate ESR36 in ovarian cells of Hx hamsters beyond a modest increase. Consistent with antral follicle formation, serum levels of E increased following the FSH treatment and levels increased further when hamsters were treated with FSH plus LH (Fig. 6A) thus validating that gonadotropin doses were physiological. LH alone did not upregulate serum E levels (Fig. 6A). Whereas LH alone or with FSH increased serum levels of progesterone, FSH had no effect (Fig. 6B). E or P treatment resulted in higher serum levels of respective steroid hormone (Figs. A and B).

### Phenobarbital suppression of the gonadotropin surge and ovarian ESR36 expression

**Figure 7:**
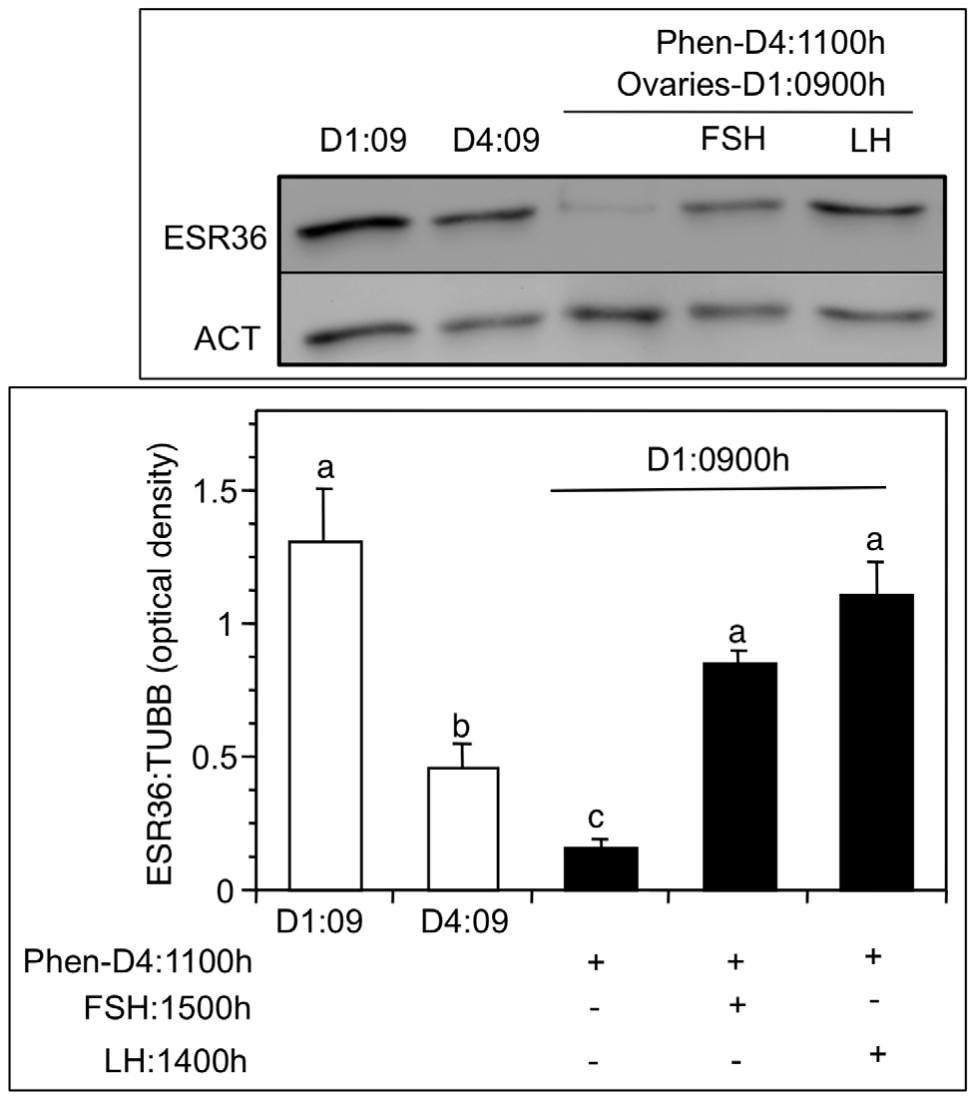
Preovulatory gonadotropin surges are responsible for the increase in ovarian ESR36 expression at D1:0090h. Hamsters were injected with 100 mg/kg body weight phenobarbital at D4∶1100 h to block the preovulatory surges of LH and FSH, as well as the postovulatory surge of FSH. LH was injected at D4∶1400 h or FSH was injected at D4∶1500 h to mimic the gonadotropin surge separately. (Top) a representative immunoblot showing the ESR36 and ACT (actin) expression in untreated ovaries at D1∶0900h and D4∶0900 h and in phenobarbital treated ovaries, and (bottom) mean optical density ± SEM of the ratio of ESR36: ACT of three separate samples. *p*<0.05, bars with a different letter.

The rationale was to determine if the gonadotropin surges were responsible for the increase in ESR36 expression at D1∶0900 h. Phenobarbital (phen) treatment at D4∶1100 h resulted in nearly complete suppression (*p*<0.001) of ovarian ESR36 expression at the next D1∶0900 h compared to untreated hamsters (Fig. 7). The decline was even lower than that observed at D4∶0900 h (Fig. 7). Phen did not affect the levels of ovarian ESR36 at D4∶1700 h, which was already low (data not shown). However, exogenously administered hormone mimicking the FSH or LH surge in hamsters treated with phenobarbital at D4∶1100 h restored ovarian ESR36 levels (Fig. 7).

## Discussion

The results of this study provide the first evidence that ESR36 is expressed differentially in ovarian cells during the estrous cycle concurrent with follicular development and changes in the levels of gonadotropins. Furthermore, the results also suggest that the ESR36 is expressed only in the plasma membrane of hamster ovarian cells, and is distinct from the ESR1 in size as validated by the specificity of the ESR36 antibody. It is evident that the expression of ESR36 is regulated directly by gonadotropins, while ovarian steroid hormones play negligible role. ESR36 is the second membrane ESR that we have discovered in the hamster ovary. The other one is GPER [36], which is expressed more in the interstitial cells and has relatively lower level of expression [19]. Using HEK293 and SKBR-3 cell lines overexpressing only ESR36 but no ESR1, [37] identified a single high affinity, saturable and low-capacity estrogen binding site in both cell lines. Therefore, it stands to reasons that ESR36 in normal ovarian cells is capable of binding estrogen for mediating the non-genomic action of estrogen.

The presence of ESR36 and GPER in ovarian plasma membrane suggests that these two receptors may mediate the non-genomic action of estrogen spatially and differentially. This contention is supported by the spatial distribution of ESR36 and GPER [19] and the expression pattern of these two receptors during the estrous cycle (present study) [19]. The existence of ESR1 and ESR36 in different cellular compartments without any overlap suggests strongly that ESR1 is not the membrane estrogen receptor at least in the hamster ovary. ESR36 is expressed predominantly in the membrane of Hec1A and MCF7 cell lines [22]; however, cytoplasmic and nuclear localization have also been documented in HEK293 cells overexpressing ESR36 [22]. In hamster ovarian cells, the endogenous ESR36 is present only in the cell membranes. This discrepancy may well be due to endogenous expression in ovarian cells versus the overexpression from a transgene in cell lines. The presence of several potential myristoylation sites in the human ESR36 leads to the assumption that the membrane localization of ESR36 is achieved by post-translational modification. The binding of ESR36 to E-affinity matrix suggests strongly that ESR36 is capable of binding the natural ligand in cell-free system. Because of the presence of both ESR36 and GPER in the ovary, it can be speculated that non-genomic action of E may play important role in preantral and early antral follicular development, whereas the maturation of follicular cells in antral follicles requires ESR1 action. Preantral and antral follicles develop in Esr1 null mice, but antral follicles become atretic afterwards [7]. The presence of an Esr1 transcript variant in Esr1 null mice has been reported [38], but if it represents ESR36 is not known. E has been shown to activate ERK [22] and Akt [26] via ESR36 in HEK293 and Hec1A cells.

The marked difference in the expression pattern of ESR36 compared to ESR1 and ESR2 in hamster follicular cells during the estrous cycles [23] suggests that E may regulate follicular cell functions throughout development via the non-genomic as well as genomic action based on the stages of follicular development. Significant ESR36 expression in the theca and interstitial cells also suggests possible extra follicular functions. The drastic fall in ESR36 expression at D4∶0900 h and D4∶1600 h when granulosa cells of preovulatory follicles are highly functional suggest that the non-genomic action of E is necessary for granulosa cell maturation; however, once the phase is over, the non-genomic action of E may not be needed for follicular cell functions. However, functional studies are needed to examine the speculation.

The upregulation of ESR36 expression by either FSH or LH but not by E or P suggests that gonadotropins directly control ESR36 expression although each gonadotropin is expected to affect specific target cell types in the ovary. Downregulation of ESR36 expression in the ovaries of phenobarbital-treated hamsters at D1∶0900 h and its reversal by gonadotropin replacement provide strong evidence that the preovulatory gonadotropin surges are responsible for the postovulatory rise in ovarian ESR36 levels. In contrast, the second FSH surge may play a limited role in ovarian ESR36 expression at D1∶0900 h because the injection of FSH at D4∶2200 h to phenobarbital-treated hamsters does not rescue the expression (data not shown). It is possible that upregulation of ESR36 protein levels by gonadotropins may require longer time. The marked increase in ESR36 expression in the non-granulosa cells in FSH-treated hamsters may be due to factors produced by the granulosa cells. FSH stimulates the secretion of a variety of growth factors and cytokines by the granulosa cells in many species including the hamster [39]–[41]. Because ESR1 downregulates ESR36 expression (30, 31) it is not surprising that exogenously added E fails to alter already low levels of ESR36 in Hx hamsters. The remarkable decrease in ESR36 expression at D4∶0900 h and a marked increase following the FSH injection in Hx hamsters further support the stimulatory role of FSH. In cyclic hamsters, FSH levels start to decrease from Day 1 morning and reach low levels by the end of Day 2 while LH levels remain low throughout [42]. Therefore, it is apparent that deficiency of gonadotropins, especially FSH, but not ovarian steroids is the cause of decrease in ESR36 in ovarian cells. The results of phenobarbital-treated hamsters further support this contention.

In summary, the results of the present study provide the first evidence that ESR36 is a transmembrane estrogen receptor expressed in hamster ovarian follicular and non-follicular cells, except the oocytes, and it is distinct from the ESR1. The expression of ESR36 is directly regulated by FSH and LH while ovarian steroid hormones have negligible role. The unique expression pattern of ESR36 during the estrous cycles suggests that the non-genomic effect of estrogen via ESR36 as well as GPER may spatially and temporally regulate granulosa cell maturation as follicles develop.
